# Contribution of Chromosomes 1H^*ch*^S and 6H^*ch*^S to Fertility Restoration in the Wheat msH1 CMS System under Different Environmental Conditions

**DOI:** 10.1371/journal.pone.0121479

**Published:** 2015-07-20

**Authors:** Almudena Castillo, Cristina Rodríguez-Suárez, Azahara C. Martín, Fernando Pistón

**Affiliations:** 1 Departamento de Mejora Genética Vegetal, Instituto de Agricultura Sostenible—Consejo Superior de Investigaciones Científicas, (IAS-CSIC), Córdoba, Spain; 2 Crop Genetics, John Innes Centre, Norwich, United Kingdom; Sabanci University, TURKEY

## Abstract

Exploiting hybrid wheat heterosis has been long pursued to increase crop yield, stability and uniformity. Cytoplasmic male sterility (CMS) systems based in the nuclear-cytoplasmic incompatible interactions are a classic way for hybrid seed production, but to date, no definitive system is available in wheat. The msH1 CMS system results from the incompatibility between the nuclear genome of wheat and the cytoplasmic genome of the wild barley *Hordeum chilense*. Fertility restoration of the CMS phenotype was first associated with the disomic addition of the short arm of chromosome 6H from *H. chilense*. In further studies it was observed that chromosome arm 1H^*ch*^S was also implicated, and the combination of genes in both chromosome arms restored fertility more efficiently. In this work we aim to dissect the effect of each chromosome in fertility restoration when combined in different genomic backgrounds and under different environmental conditions. We propose a model to explain how restoration behaves in the msH1 system and generate valuable information necessary to develop an efficient system for hybrid wheat production.

## Introduction

The discovery of heterosis or hybrid vigor and its exploitation in modern breeding programs is one of the most important advances in plant breeding in the last century. Hybrid varieties have led to a large increase in yield and uniformity in many crop species [1]. Cytoplasmic male sterility (CMS) is a condition under which a plant is unable to produce functional pollen due to the interaction between the cytoplasm and the nuclear genomes. It represents a valuable tool in the production of hybrid seed in self-pollinated crop species, and it has been successfully used in many cropping systems including rice, maize, sunflower and rye among others [2, 3]. However, despite multiple attempts, no optimum system for hybrid production has been successfully established in wheat [4].

The new CMS system msH1 was first reported in bread wheat (*Triticum aestivum* L.) by [5]. This system uses the cytoplasm of *Hordeum chilense Roem. et Schultz.* (2n = 2x = 14, H^*ch*^H^*ch*^), a diploid wild barley native to Chile and Argentina which possesses some traits potentially useful for wheat breeding [6–8]. Moreover this species exhibits high crossability with other members of the *Triticeae* tribe [9, 10]. Fertility restoration of the CMS phenotype caused by the *H. chilense* cytoplasm was first associated with the addition of the short arm of chromosome 6H^*ch*^from *H. chilense* [5]. An extra acrocentric chromosome, named H^*ch*^ac, capable of restoring male fertility even more efficiently than 6H^*ch*^S was observed while testing this system in other wheat backgrounds [11]. The acrocentric chromosome restored fertility even in monosomic condition, in contrast to chromosome 6H^*ch*^S which only fully restored fertility when present in homozygosis. Further studies of this acrocentric chromosome with molecular markers and cytological techniques, demonstrated that itcomprises fragments of the short arms of chromosomes 6H^*ch*^ and 1H^*ch*^ [12].

Fertility restoration is genetically very complex as, though mainly controlled by restorer genes, it is also affected by the genomic background and the environmental conditions. Factors such as temperature, photoperiod and light intensity are thought to be of primary importance for the development of fertility. In fact, the influence of environment on the expression of CMS and its fertility restoration has been observed in a large number of crops species [3, 13]. Photoperiod and temperature have also been reported to influence wheat pollen viability [14, 15]. Therefore, for the application of hybrid wheat technology, the study of the interaction between environmental factors and fertility restoration is absolutely necessary.

To date we know that restorer genes in the msH1 system are located in the 6H^*ch*^S and 1H^*ch*^S chromosomes, and that it is the combination of both, which completely restores male fertility [12, 16]. The objective of this work is to analyze the interaction between different genomic combinations of these two chromosomes and different environmental conditions, in order to establish their contribution to fertility restoration in the msH1 system.

## Materials and Methods

### Plant material

Crosses using lines shown in Table 1 were carried out in april 2012 in Córdoba, Spain, to obtain different genomic combinations involving the short arms of chromosomes 1 (1H^*ch*^S)and 6 (6H^*ch*^S) of *H. chilense* in an alloplasmic wheat background with *H. chilense* cytoplasm. The genetic constitution of each parental line is that described by their authors: lines T21A1H_1_S and T552 were kindly provided by Steve Reader, JIC, Norwich, UK; lines T218 and T593 are described in [5]; and lines T650 and T236 are described in [11, 16], respectively. The progeny of the crosses was cytologically screened by somatic chromosome counting to confirm that seeds came from crosses and not from self-fertilizations. For this, root tips of 1-cm length were collected from germinating seeds and pre-treated for 4 h in an aqueous colchicine solution (0.05%) at 25°C, fixed in a freshly prepared 3 absolute ethanol:1 glacial acetic acid (*v*/*v*) mixture and stained by the conventional Feulgen technique.

**Table 1:** Description of the plant material used to obtain the different alloplasmic lines.

Line	St ab.	Germplasm	n	Conf.	Fertility
T21	CS	*T. aestivum* cv. Chinese Spring in CS cytoplasm	42	21”	Fertile
T218	(H1)CS	*T. aestivum* cv. Chinese Spring in H1 cytoplasm	42	21”	Male sterile
T236	(H1)T26	*T. aestivum* cv. T26 in H1 cytoplasm	42	21”	Male sterile
T552	CS-H^*ch*^DT1H^*ch*^S⋅1BL	*T. aestivum* cv. Chinese Spring*H. chilense* double translocation 1H^*ch*^S⋅1BL in CS cytoplasm	42	20” + 1”T1H^*ch*^S⋅1BL	Fertile
T650	(H1)CS-H^*ch*^ DT6H^*ch*^S⋅6DL	*T. aestivum* cv. Chinese Spring-*H. chilense* double translocation 6H^*ch*^S⋅6DL in H1 cytoplasm	42	20” + 1”T6H^*ch*^S⋅6DL	Fertile
T593	(H1)CS-H^*ch*^ DtA6H^*ch*^S	*T. aestivum* cv. Chinese Spring-*H. chilense* ditelosomic addition 6H^*ch*^S in H1 cytoplasm	42+t”	21” + t”6H^*ch*^S	Fertile
T21A1H_1_S	CS-H^*ch*^ DtA1H^*ch*^S	*T. aestivum* cv. Chinese Spring*H. chilense* ditelosomic addition 1H^*ch*^S in CS cytoplasm	42+t”	21” + t”1H^*ch*^S	Fertile

n: chromosome number; Conf: chromosome configuration; St ab.: standard abbreviation.

### Experimental design

At least three F1 plants of each cross were sown in pots in November 2013, in Córdoba, in order to assess the effects of the genetic constitution and the environment on fertility restoration. Each of the different alloplasmic F1 individuals was split when they were three weeks old to generate two clones per plant. One of the clones was grown in a greenhouse and the other was transplanted into the field The experimental design was a completely randomized design replicated in two environmental conditions, ‘field’ and ‘greenhouse’.

Seed set after anthesis was used as the criterion for assessing male fertility and sterility. Seed set of all plants was evaluated by two methods: (1) by counting the number of grains per lateral flower in 20 flowers located in the middle of every spike; and (2) by counting total number of grains per total number of flowers in the spike. Fertility indices for both estimators were calculated as the ratio of the number of grains per flowers (number of grain/number of flowers), where a value of 1 means total fertility and 0 means total sterility. In both cases an average number of five flowers per spikelet was considered. Along with the seed set of all the spikes in every plant, flowering date, day length, and minimum (Tmin), mean (Tmean) and maximum (Tmax) temperatures at anthesis were recorded.

### Statistical analysis

All analyses were conducted with the statistical software R version 3.0.2 [17]. In order to determine the usefulness of the first estimator of fertility, a correlation analysis between both parameters was performed.

Fertility data scored with the first method were adjusted to linear models including the following explanatory variables: Location (‘field’/‘greenhouse’), flowering date, day length and minimum, mean and maximum temperatures. Data were adjusted to a linear model with the function ***lm*** and factors effects were checked by an analysis of variance with the function ***anova***. The normality and heteroscedasticity assumptions were tested by plotting the residuals versus the predicted values and Q-Q plots.

The linear model that best explained the observed variation was selected. Briefly, in a first step all the variables and factors were included (saturated model) with all two-way interactions between them. The saturated model was then reduced by the elimination of factors and covariates with non significant effect.

The differences between alloplasmic lines were assessed using post hoc multiple-comparison test (function ***glht***, package ***multcomp*** [18]. The method used for multiple-comparison was a Tukey contrast with a *p-values* adjustment type “free” (see package ***multcomp*** for more information). The least-squares (LS) means were calculated with the package *lsmeans* [19].

All R code and data used in this work are freely available at https://github.com/fpiston/Paper_CMStmp.

## Results

The different crosses carried out to obtain the alloplasmic lines with the different genetic combinations of chromosomes 1H^*ch*^S and 6H^*ch*^S are shown in Table 2. Alloplasmic line T218 was pollinated with T21A1H_1_S, T552 and T650 to obtain the monosomic addition of 1H^*ch*^S, and the monosomic translocations of 1H^*ch*^S⋅1BL and 6H^*ch*^S⋅6DL, respectively. Line T236 was pollinated with T552 to obtain the monosomic translocation of 1H^*ch*^S⋅1BL in a different wheat background. Line T593 was pollinated with T21, T552 and T21A1H_1_S to obtain the monosomic addition of 6H^*ch*^S in the first case, the monosomic translocation of 1H^*ch*^S⋅1BL with the monosomic addition of 6H^*ch*^S in the second, and the monosomic additions of 1H^*ch*^S and 6H^*ch*^S in the latest. The double monosomic translocation of 1H^*ch*^S⋅1BL and 6H^*ch*^S⋅6DL was obtained by pollinating line T650 with T552.

**Table 2:** Different genomic combinations involving 1H^*ch*^S and 6H^*ch*^S chromosomes arms obtained in this work.

Line	Origin	St ab.	Germplasm	n	Conf.
T218T21A1H_1_S	T218 × T21A1H_1_S	(H1)CS-H^*ch*^ MtA1H^*ch*^S	*T. aestivum* cv. Chinese Spring–*H. chilense* monotelosomic addition 1H^*ch*^S in *H. chilense* cytoplasm	42+t’	21”+t’1H^*ch*^S
T218T552	T218 × T552	(H1)CS-H^*ch*^ T1H^*ch*^S⋅1BL	*T. aestivum* cv. Chinese Spring–*H. chilense* translocation 1H^*ch*^S⋅1BL in *H. chilense* cytoplasm	42	20”+1’1B+1’ T1H^*ch*^S⋅1BL
T218T650	T218 × T650	(H1)CS-H^*ch*^ T6H^*ch*^S⋅6DL	*T. aestivum* cv. Chinese Spring–*H. chilense* translocation 6H^*ch*^S⋅6DL in *H. chilense* cytoplasm	42	20”+1’6D+1’ T6H^*ch*^S⋅6DL
T236T552	T236 × T552	(H1)T26-H^*ch*^ T1H^*ch*^S⋅1BL	*T. aestivum* cv. T26–*H. chilense* translocation 1H^*ch*^S⋅1BL in *H. chilense* cytoplasm	42	20”+1’1B+1’ T1H^*ch*^S⋅1BL
T593T21	T593 × T21	(H1)CS-H^*ch*^ MtA6H^*ch*^S	*T. aestivum* cv. Chinese Spring–*H. chilense* monotelosomic addition 6H^*ch*^S in H1 cytoplasm	42+t’	21”+t’6H^*ch*^S
T593T21A1H_1_S	T593 × T21A1H_1_S	(H1)CS-H^*ch*^ MtA6H^*ch*^S MtA1H^*ch*^S	*T. aestivum* cv. Chinese Spring–*H. chilense* monotelosomic addition 6H^*ch*^S monotelosomic addition 1H^*ch*^S in H1 cytoplasm	42+t”	21”+t’6H^*ch*^S+t’1H^*ch*^S
T593T552	T593 × T552	(H1)CS-H^*ch*^ MtA6H^*ch*^S T1H^*ch*^S⋅1BL	*T. aestivum* cv. Chinese Spring–*H. chilense* monotelosomic addition 6H^*ch*^S translocation 1H^*ch*^S⋅1BL in H1 cytoplasm	42+t’	21”+1’BL+1’T1H^*ch*^S⋅1BL+1’t6H^*ch*^S
T650T552	T650 × T552	(H1)CS-H^*ch*^ T6H^*ch*^S⋅6DL T1H^*ch*^S⋅1BL	*T. aestivum* cv. Chinese Spring–*H. chilense* translocation 6H^*ch*^S⋅6DL translocation 1H^*ch*^S⋅1BL in H1 cytoplasm	42	21”+1’6D+1’T6H^*ch*^S⋅6DL 1’BL+1’T1H^*ch*^S⋅1BL

n: chromosome number; Conf: chromosome configuration; St ab: standard abbreviation.

### Fertility evaluation

**Fig 1:**
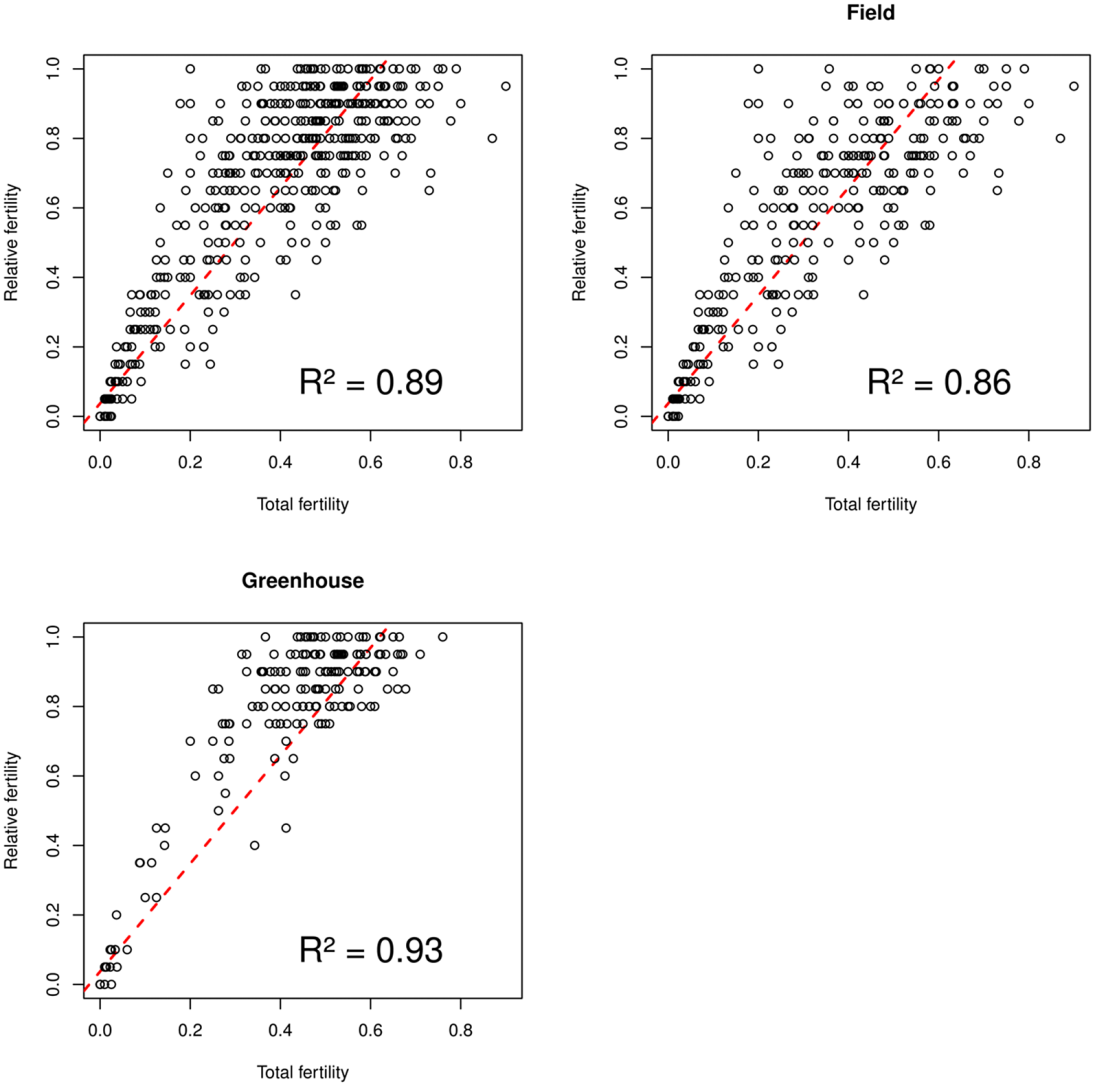
Correlation between the two fertility estimators using A) total data, B) ‘field’ data and C) ‘greenhouse’ data. A fertility value of 1 means complete fertility and 0 means complete sterility.

The relationship between the two indices used for evaluating the fertility 1) count of the number of grains produced by the 20 central flowers in the spike, and 2) count of the total number of grains per spike was analyzed. A strong correlation between the two fertility estimators was found when using total data (R^2^ = 0.8883). As shown in Fig 1, it was even higher when only ‘greenhouse’ location data were considered (R^2^ = 0.9296). As also observed in Fig 1, correlation was higher for low fertility values and decreased as fertility values increased.

### Effects of different factors on fertility: fitting a model

**Fig 2:**
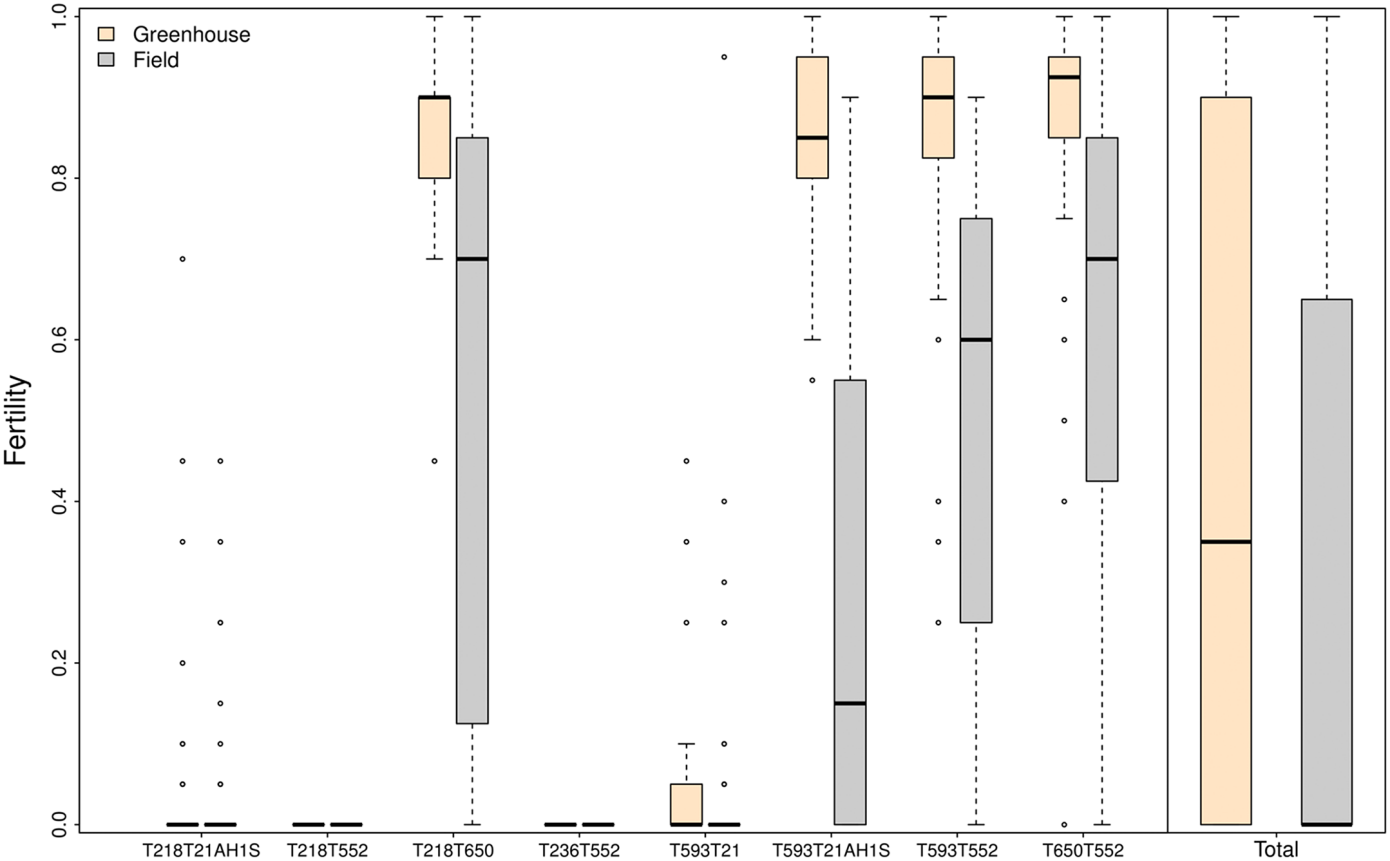
Fertility of the genotypes studied in two different locations (‘greenhouse’ and ‘field’). A fertility value of 1 means complete fertility and 0 means complete sterility.

For elucidating the best model the reduced model obtained was: ‘Fertility ∼ Genotype + Location + Tmean + Genotype:Location + Genotype:Tmean + Location: Tmean’. ANOVA table for this model, named lm1r1, is shown in Table 3. In this reduced model the variables day-length, minimum and maximum temperatures were removed since none of them had a significant effect on fertility. Genotype (60.5% of explained variance) was the factor with the greatest effect on fertility. Lines T218T21A1HS, T218T552, T236T552 and T593T21 were male sterile in both the greenhouse and the field, considering sterile those plants with an average fertility below 0.2 in all environments. The other four lines T650T552, T218T21A1H_1_S, T593T552 and T650T552 weremale fertile at some degree depending on the growth location, indicating a significant interaction Genotype:Location (Fig 2).

**Table 3:** ANOVA analysis for the model ‘lm1r1’. In this reduced model the variables day-length, and minimum and maximum temperatures were removed since none of them had a significant effect on fertility.

	Df	Sum Sq	% Variance	*P-value*
Genotype	7	88.733	60.53	< 2.2e-16
Location	1	8.569	5.85	< 2.2e-16
Tmean	1	0.992	0.68	2.436e-07
Genotype×Location	7	9.743	6.65	< 2.2e-16
Genotype×Tmean	7	2.214	1.51	2.764e-10
Location×Tmean	1	1.536	1.05	1.551e-10
Residuals	949	34.799		

Df: degree of freedom; Sum Sq: sum of squares; % Variance: percent of explained variance.

A robust fertility is defined by a high degree of fertility and slight variations among individuals (low dispersion of the individual’s fertility values). Within the fertile genotypes differences in robustness were detected, with genotype T218T21A1H_1_S showing less robustness in the ‘greenhouse’ than in the ‘field’. ‘Location’ is the second factor in the amount of explained variance. Plants grown in the ‘greenhouse’ had a higher and more robust fertility than those grown in the ‘field’ (Fig 2).

**Fig 3:**
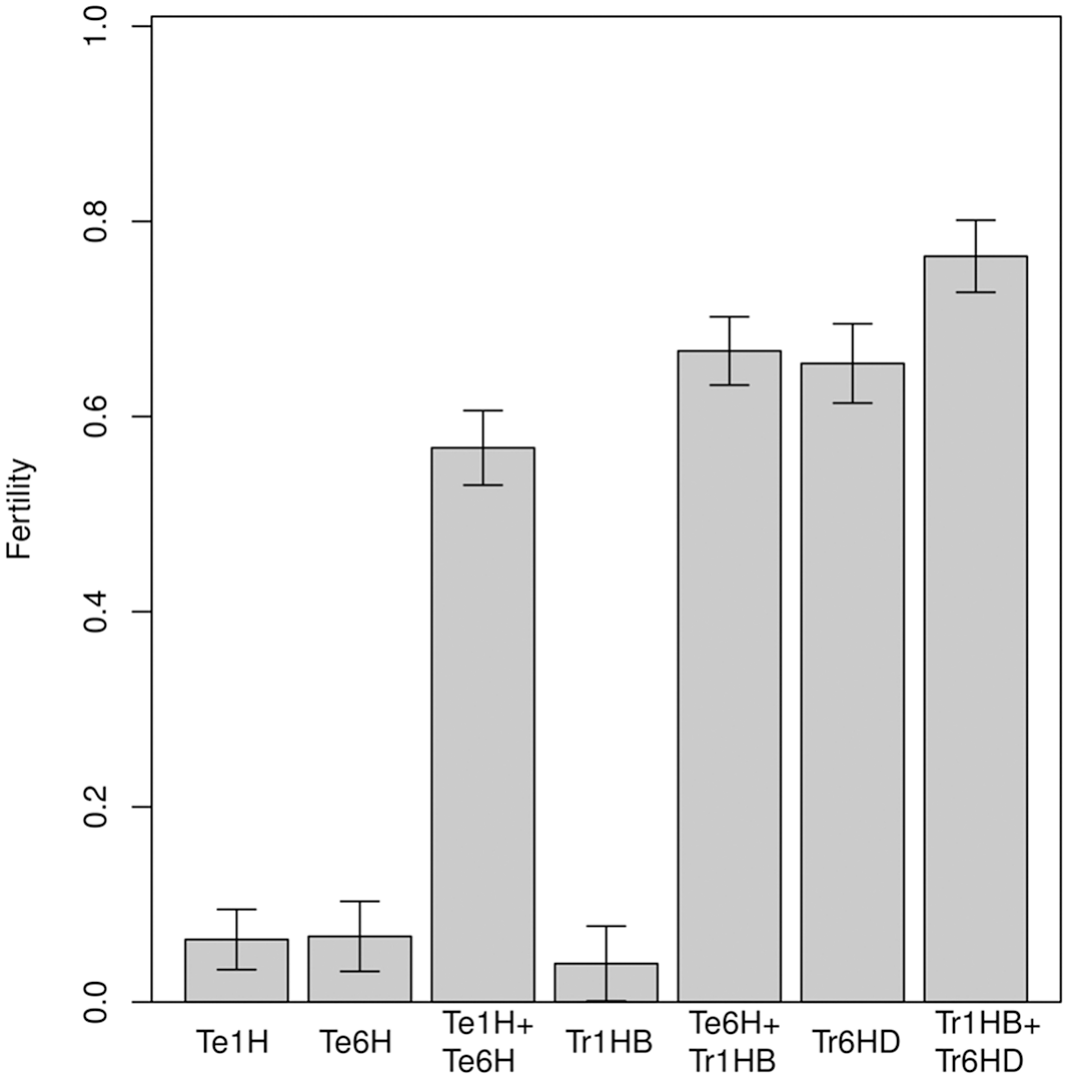
Fertility values adjusted according to the model ‘lm1r1’ by ‘Genotype’, ‘Tmean’, and growth location. Grey shaded boxes show fertile genotypes.

Another factor with significant effect on fertility was ‘Tmean’. In general, fertility increased with temperature but in some cases this relationship was unclear due to interactions with factors ‘Genotype’ and ‘Location’ (Table 3). For that reason the effect of temperature on fertility by ‘Genotype’ and ‘Location’ was studied separately. Fig 3 shows the values of fertility adjusted according to the model ‘lm1r1’ by ‘Genotype’, ‘Tmean’, and ‘Location’. In the case of the field-grown plants, fertility increases with temperature in all genotypes, mainly T218T650, T593T552 and T650T552. In the case of the plants grown in the ‘greenhouse’, the sterile genotypes show a decrease in fertility with temperature; and the fertile genotypes increases with the temperature in the T218T650 and T593T552 genotypes, and decreases slightly in the T218T21A1H_1_S and T650T552 genotypes. These differences in fertility patterns with respect to temperature and genotype cause the significant interaction between factors ‘Genotype’, ‘Tmean’ and ‘Location’.

**Fig 4:**
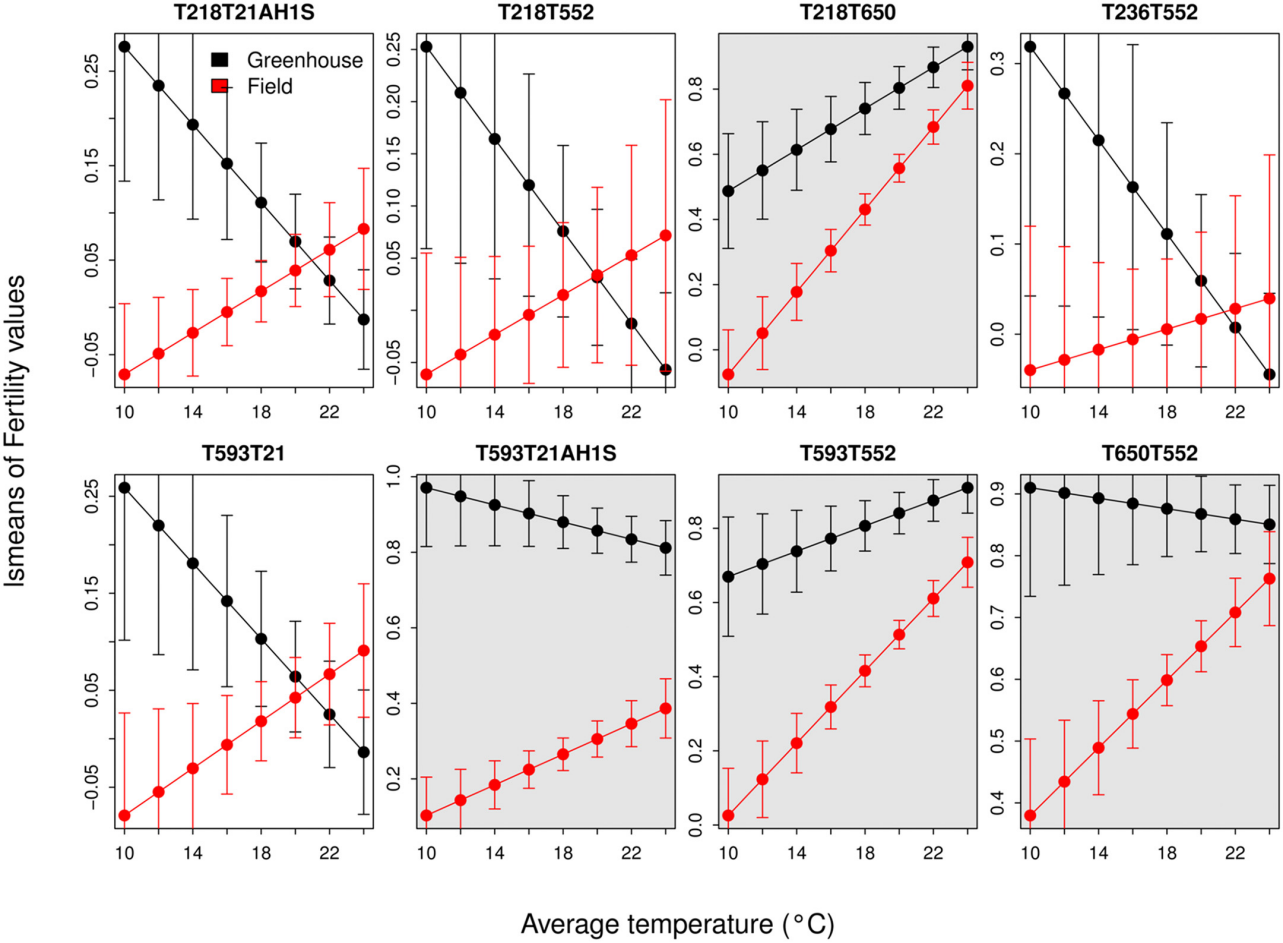
Fertility of the different genetic combinations involving 1H^*ch*^S and 6H^*ch*^S chromosomes arms. Te1H: monotelosomic addition 1HchS; Te6H: monotelosomic addition 6HchS; Tr1HB: translocation 1HchS⋅1BL; Tr6HD: translocation 6HchS⋅6DL. A fertility value of 1 means complete fertility and 0 means complete sterility.

To dissect the factor ‘Genotype’, the effect of each chromosome combination was analyzed separately. A new model, called lm1r1C, was adjusted where the factor ‘Genotype’ was eliminated and the following factors were incorporated: a) monotelosomic addition 1HS (Te1H), b) monotelosomic addition 6HS (Te6H), c) translocation 1HS⋅1BL (Tr1HB) and, d) translocation 6HS⋅6DL (Tr6HD). The model formula is: ‘Fertility ∼ Location + Tmean + Te1H + Te6H + Tr1HB + Tr6HD + Te1H:Te6H + Te6H:Tr1HB + Tr1HB:Tr6HD + Location:Tmean + Location:Te1H + Location:Te6H + Location:Tr1HB + Location:Tr6HD + Tmean:Te1H + Tmean:Te6H + Tmean:Tr1HB + Tmean:Tr6HD’. To compare the effect of each genetic combination involving 1H^*ch*^S and 6H^*ch*^S chromosomes on fertility, the LS means from the model lm1r1C were calculated with confidence intervals at a significance level of 0.05 (Fig 4). The monotelosomic addition of 1H^*ch*^S, the monotelosomic addition of 6H^*ch*^S and the translocation 1HS⋅1BL did not increase fertility. Conversely, the monotelosomic additions of both 1HS and 6HS significantly increased fertility. The greatest suscess in the restoration of fertility was achieved when the translocation 6HS⋅6DL was present and there were no significant differences with or without the translocation 1HS⋅1BL. A much larger increase was produced when both 6HS⋅6DL and 1HS⋅1BL translocations were combined in a single line. Although the monotelosomic addition of 1HS or the translocation 1HS⋅1BL translocation did not increase fertility by themselves they have a synergistic effect on increasing fertility when they are together with the monotelosomic addition of 6HS or with the translocation 6HS⋅6DL.

**Fig 5:**
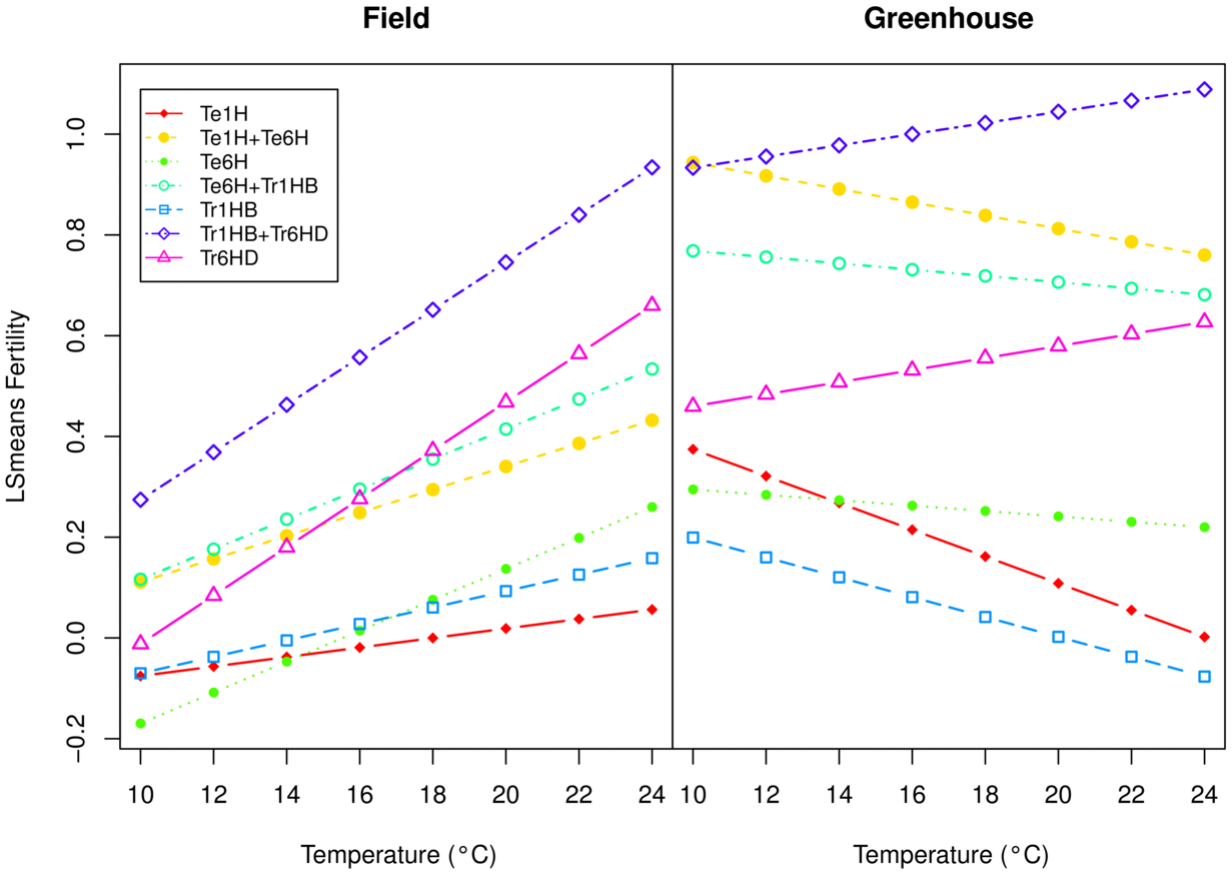
Fertility of different chromosome combinations using LSMEANS with respect to the temperature in the two growing locations (‘Field’ / ‘Greenhouse’). The LS means were calculated from the model ‘lm1r1C’ where the factor ‘Genotype’ was eliminated to analyze the effect of each chromosome combination separately.

Fig 5 shows the fertility values of different chromosome combinations adjusted using LS means with respect to the temperature at the two locations, ‘field’ and ‘greenhouse’. The LS means were calculated from the model lm1r1C described above. For plants grown in the ‘field’, all chromosome combinations have a positive fertility response to increasing temperatures, although translocation 6HS⋅6DL have a greater response, as evidenced by the curve slope (Fig 5). Conversely, in the ‘greenhouse’ the only genotype with a positive response to temperature is 6HS⋅6DL translocation.

## Discussion

Since the msH1 CMS system was first described, great effort has gone into identifying the *H. chilense* chromosome arm responsible for fertility restoration. The involvement of chromosomes 1H^*ch*^S and 6H^*ch*^S in fertility restoration has been reported in previous works of the group [5, 11, 12, 16]. In this work we go one step further and evaluate the fertility restoration ability of different combinations of these two chromosomes (translocation and/or addition lines) in different backgrounds and environmental conditions.

Concurrently, two different methods of fertility estimation were investigated, the number of grains produced by 20 central flowers of the spike and the total number of grains per spike. The correlation between them was very high when it is present. Because there are no large differences between the fertility estimation by both methods, we propose the method of the 20 flowers for its speed and ease of evaluation.

The results presented in this work show that the most important factor controlling fertility restoration in the msH1 CMS system is the genotype, in particular the chromosome arm 6H^*ch*^S, as expected based on our previous knowledge of the system. Genotypes with the monosomic telocentric addition of either chromosome 1H^*ch*^S or 6H^*ch*^S do not show an increase in fertility. However when both chromosome arms are present as telocentric additions, the level of fertility is significantly increased. Higher fertility levels are shown by those genotypes harbouring the 6H^*ch*^S⋅6DL translocation. Conversely lines with the translocation 1H^*ch*^S⋅1BL do not show increase in fertility. However, lines that present both translocations 6H^*ch*^S⋅6DL and 1H^*ch*^S⋅1BL show a much larger increase.

Although the region of chromosome 1H^*ch*^S itself does not increase fertility, it enhances the positive effect of chromosome 6H^*ch*^S when is present translocated with a wheat chromosome. This implies that the major gene/s controlling fertility restoration are located on the short arm of chromosome 6H^*ch*^ but that an enhancer gene/s are also present in the 1H^*ch*^S chromosome. These results are consistent with previous observations on the restoration ability obtained with the H^*ch*^ac chromosome. It has been recently shown that H^*ch*^ac is a kind of zebra-like chromosome conformed by fragments of the 6H^*ch*^S and 1H^*ch*^S arms, indicating again that restoration is enhanced by the interaction of genes in both chromosomes [12].

The location where plants are grown is the second factor affecting fertility restoration. In this work, plants grown in the greenhouse have higher and more stable fertility values (less dispersion of the fertility values) than those grown in the field. Plants grown in the field are exposed to more extreme environmental conditions, which may negatively affect fertility. However, in the greenhouse, environmental conditions are damper and more stable, which might promote a more stable fertility as well. The factor ‘Location’ could then be masking the effect of other factors; therefore a deeper examination of the environmental conditions is needed to find other abiotic elements affecting the fertility restoration in the msH1 system, apart from those studied in this work.

Lastly, the average temperature at anthesis is significantly associated with fertility. Overall fertility of plants grown in the field increased with an increase in temperature. On the other hand, in the greenhouse, fertility does not change with temperature, or it even decreases as temperature increases. An exception is plants with the translocation 6H^*ch*^S⋅6DL. Plants with this translocation have a positive response to temperature in both environments showing male sterility at low temperatures and restored fertility at high temperatures. Furthermore, the increase of fertility by degree of average temperature is greater than in the rest of the chromosomal combinations studied in this work. This could be explained by the existance of a gene/s located on chromosome 6DS with a negative effect on fertility, and that is absent in the translocation event (6H^*ch*^S⋅6DL). Thermo-sensitive cytoplasmic male sterility lines have been frequently observed in other CMS systems. Indeed, this is the basis for the development of ‘two line hybrid systems’ in which temperature at anthesis determines fertility phenotype [20, 21].

We have observed some association between photoperiod and plant location; however, our results demonstrated that fertility was not influenced by day-length as in other CMS systems [20]. For this reason, we cannot reliably separate the effect on fertility of factors ‘Location’ and ‘Length day’ and more studies on the effect of photoperiod on fertility in wheat with *H. chilense* cytoplasm with and without nuclear genes restorers are needed.

In conclusion, the main factor explaining fertility restoration in the msH1 system is the genotype. The combination of both 6H^*ch*^S⋅6DL and 1H^*ch*^S⋅1BL translocations is the best one for fertility restoration considering all the situations tested. In any case, the presence of genes from both 6H^*ch*^S and 1H^*ch*^S is necessary to achieve full fertility restoration. It has been reported that the expression of partial restorer alleles is more strongly affected by environmental conditions than the expression of fully restoring and non-restoring alleles [22]. Thus fully fertile plants may change their degree of fertility in a different location or environment. Fertility responses in the greenhouse were more uniform and consistenthan in the field and then, fertility restoration in the msH1 system is more predictable in a controlled environment where temperature must be also taken into consideration. In general, plants seem to deal with translocations better than with telocentric additions, suggesting a less negative impact on the overall condition of the plant of the formersand, therefore, in fertility.
